# Higher Local Food Consumption Is Associated with Higher Adherence to the Mediterranean Diet and Better Healthy Aging: Results of the DIAPELH Study

**DOI:** 10.3390/nu17182975

**Published:** 2025-09-17

**Authors:** Alexandra Foscolou, Giannoula Nikolaou, Trisevgeni Pratti, Antigone Kouskouti, Vasiliki Kanellaki, Eirini Machaira, Izabella Bekari, Evanthia Chalari, Aristea Gazouli, Aristea Gioxari

**Affiliations:** Department of Nutritional Science and Dietetics, School of Health Sciences, University of the Peloponnese, Antikalamos, 24100 Kalamata, Greece; alexandra.foscolou@go.uop.gr (A.F.); t.pratti@go.uop.gr (T.P.); a.kouskouti@go.uop.gr (A.K.);

**Keywords:** local foods, Mediterranean diet, healthy aging, successful aging, older adults

## Abstract

**Background/Objectives**: Sustainable dietary patterns in geriatrics have gained considerable attention. In this cross-sectional study, we investigated whether higher consumption of locally produced foods is associated with higher adherence to the Mediterranean diet and with better healthy aging status among Greek older adults. **Methods**: Sociodemographic, anthropometrical, lifestyle, dietary, cognitive, and mental characteristics assessed through validated questionnaires and procedures, were assessed. Participants (N = 449) were divided into three local food consumption groups/tertiles: “high” (≥45% of total dietary intake), “moderate” (27–44%) and “low” (≤26%). MedDietScore (0–55) was used to assess the level of adherence to the Mediterranean diet and SAI (0–10) to assess healthy aging. **Results**: Older adults of the “high” group showed an increase of 0.817 units in the SAI index compared to older adults of the “low” group. Additionally, high local food consumption was also associated with higher SAI levels (b = 0.493, *p* = 0.007) compared to the “moderate” group. Among older individuals whose diet comprises over 45% local foods, an increase of approximately 2.8 and 1.95 units in the MedDietScore was detected when compared to the “low” and “moderate” groups. **Conclusions**: Higher consumption of local foods, and more specifically consuming local foods in more than 45% of the total dietary intake, is associated with greater adherence to the Mediterranean diet and better healthy aging. The results suggest that food locality may play an important role in shaping better dietary habits, health trajectories, and quality of life of older adults.

## 1. Introduction

In recent years, the scientific community has raised serious concerns about environmental and human health sustainability [1]. Several dietary patterns have been proposed as capable of promoting both environmental resilience and human well-being with the Mediterranean diet being among the most researched and scientifically validated [2,3,4]. Recognized as a model of sustainability for populations residing in Mediterranean regions [5], the Mediterranean diet has been associated with substantial health benefits and improvements in quality of life across the lifespan [6]. The Mediterranean diet is rooted in the traditional culinary practices of countries bordering the Mediterranean Sea [7]. It is characterized by a high intake of whole grains, fruits, vegetables, legumes, and potatoes, with olive oil serving as the primary source of dietary fat. The pattern also includes regular fish consumption, moderate consumption of dairy, eggs and poultry, limited consumption of red meat, and modest amounts of wine, typically consumed during meals [7].

At the same time, the concept of local foods has gained increasing importance and interest due to its relevance to environmental sustainability, cultural preservation, and nutritional quality [8,9,10]. Although definitions of locality vary, the Joint Research Centre of the European Commission used as criterion the production distance not exceeding 100 km from the point of consumption [11]. The consumption of locally produced foods has been associated with a reduced environmental footprint, support for the local economy, and improved access to fresher and less processed foods [12,13]. It should also be noted that, although in the Mediterranean region local foods are often tautological with the components of the Mediterranean diet, the two concepts are very different. For example, olive oil and vegetables—core components of the Mediterranean diet and commonly considered local products—may be produced within the region or imported from other regions of the same country or from abroad. In the first case, these products are consumed fresher, with higher preservation of key nutrients and bioactive compounds compared with foods imported from elsewhere [14].

Meanwhile, the global population is increasing rapidly, particularly due to a significant rise in the elderly demographic [15]. This trend is clearly evident in many Mediterranean countries, including Greece, where the proportion of the population aged 65 years and over was approximately 22.4% in 2020 and is projected to reach around 31.5% by 2050 [16]. Older ages are frequently accompanied by the onset of chronic diseases, functional impairments, and other conditions that require increased medical attention [15]. Consequently, promoting healthy aging has become a crucial public health objective. The concept of healthy aging is quite complex; however, current theoretical frameworks agree on the concept that it encompasses maintenance of quality of life (physical and mental functioning) and the absence of disease [17,18]. Dietary habits are essential to this process, since they profoundly affect physical health outcomes, cognitive function, and psychological well-being [19].

Although numerous studies have demonstrated the benefits of the Mediterranean diet for healthy aging, relatively few have focused on the role of food locality as a potential mediating or enhancing factor. Thus, the aim of the present work was to investigate whether higher consumption of locally produced foods is associated with higher adherence to the Mediterranean diet and with better healthy aging status among older adults in Greece. The findings are expected to provide valuable evidence to guide the design of targeted public health policies and agri-food interventions.

## 2. Materials and Methods

### 2.1. Study Design and Participants

The present cross-sectional study is part of the DIAPELH project (derived from the Greek acronym “ΔΙA.ΠΕΛ.H—Διατροφή στην Πελοπόννησο των Hλικιωμένων”) which is an ongoing study in the region of Peloponnese. Its objective is to examine the association between several socio-economical, anthropometrical, lifestyle, nutritional, functional, physical, and mental health-related characteristics with healthy aging indicators among individuals over 60 years of age in Peloponnese. The Peloponnese is a particularly interesting region from a nutritional perspective. It is the land of the olive tree, a core component of the Mediterranean diet. It is characterized by relatively fertile soils for cultivation and livestock farming, on which the economy of its inhabitants is largely based. Therefore, the Peloponnese may be considered an area of specific interest in terms of dietary habits of its inhabitants, especially older adults, who probably have preserved to a greater extent the practices and habits of earlier decades, before Western influences. Individuals were approached through written announcements (posters) in local newspapers, community centers, local shops, and healthcare facilities. Recruitment was also facilitated by collaboration with municipalities throughout the region of Peloponnese. Additionally, social media posts and digital invitations were made.

Participants of both genders, aged over 60 years, and permanent residents of the Peloponnese were included in the study, but individuals residing in care facilities or living in the Peloponnese for less than 5 years, were omitted.

### 2.2. Bioethics

The present study was approved by the School of Health Sciences Research Ethics Committee at the University of Peloponnese (Approval no 04/11 September 2023). The study was conducted in compliance with the principles of the Declaration of Helsinki. Participants were informed of the study’s aims and methods, and all gave signed informed consent before recruitment.

### 2.3. Measurements

A quantitative questionnaire and established protocols were employed to gather the requisite information, more specifically as follows:

*Sociodemographic and lifestyle characteristics*: The evaluated sociodemographic parameters were age (in years), sex (male/female), and daily walking duration (in minutes). Smoking behaviors were also evaluated; individuals were classified as “smokers” if they smoked at least one cigarette or other tobacco product daily, when the interview was taking place.

*Anthropometry*: Established protocols were employed to determine body weight and height. The measurement of body weight was conducted to the nearest 0.1 kg, while height was measured to the nearest 0.1 cm. Body mass index (BMI) was computed as body weight (kg)/height^2^ (m^2^). Waist and hip circumferences were assessed with a flexible, non-elastic tape while the individuals were unclothed. Waist circumference was assessed at the midpoint between the lowest rib and the iliac crest using an inelastic measuring tape to the nearest 0.1 cm, while hip circumference was measured between the greater trochanter and the inferior buttock level [20]. The waist-to-hip ratio was used to identify abdominal obesity.

*Dietary intake*: A validated and reliable semi-quantitative food frequency questionnaire was used to assess dietary habits [21]. The consumption of a variety of food groups and beverages, including meat and meat products, poultry, fish, milk or other dairy products, fruits, vegetables, legumes, and alcohol, was assessed weekly (never or rarely, 1–3 times/month, 1–2 times/week, daily, 3–6 times/week, daily, more than twice daily). The MedDietScore was employed to evaluate the level of adherence to the Mediterranean diet with a theoretical range of 0 to 55 [22]. Higher values indicate higher adherence. In addition, the percentage of local foods consumed by the participants was estimated based on their responses for each specific food group included in the questionnaire. During the sessions with the dieticians, participants indicated their estimation of the proportion of local products they usually consumed for each food or food group (i.e., fruits, vegetables, cereals, potatoes, legumes, milk, cheese, yogurt, nuts, olive oil, fish, red meat, poultry, alcohol, snacks, desserts, non-alcoholic beverages). Then, the mean value of these proportions and the average percentage were calculated as an overall indicator of local food consumption in their diet.

*Depression symptomatology*: A brief, self-administered Geriatric Depression Scale (GDS), translated and validated in Greek, with a total score ranging from 0 to 15, was employed to assess depressive symptoms during the last month. Increased scores indicate greater severity of depressive symptoms [23].

*Cognitive function*: The Montreal Cognitive Assessment (MoCA) test was used to assess the cognitive abilities of the older adult participants. MoCA evaluates various cognitive domains: attention and focus, executive processes, memory, language, visuoconstructive skills, conceptual reasoning, calculation ability, and orientation. MoCA scores range from 0 to 30, with higher values indicating better cognitive function [24].

*Healthy aging*: The assessment of healthy aging used a previously established and validated instrument, specifically the SAI (Successful Aging Index) index [25]. The SAI index was constituted by education (years of education), financial status (through self-reported mean income over the past three years, classified on a four-point scale: low, medium, good, and very good), physical activity (METs), BMI, depression (assessed via GDS), social activity (family or friends) participation (times/week), annual excursions, cardiovascular disease risk factors (including obesity, hypertension, hypercholesterolemia, diabetes mellitus), and adherence to the Mediterranean diet [25].

### 2.4. Sample Size Calculation and Statistical Analysis

Sample size calculation was performed, and we hypothesized a percentage frequency of the outcome factor in the population of 50% (±5), within confidence levels of 95% and a design effect of 1 (randomized sample). The minimum sample size was estimated to be 384.

Normality was tested using the Kolmogorov–Smirnov test. Categorical values are expressed as frequencies and percentages, with continuous variables as median and interquartile ranges (non-normally distributed data). Comparisons of continuous variables were performed using the Kruskal–Wallis H test, while associations between categorical variables were tested using the chi-square test. Pairwise comparisons between local food consumption groups were performed using Mann–Whitney U test. Linear regression analyses were conducted to assess the associations between (a) dummy variables for local food percentage consumption (High vs. Low, High vs. Moderate, and Moderate vs. Low) and (b) local food consumption across the eleven Mediterranean food groups, with two outcomes: the SAI, as an overall index of healthy aging, and the MedDietScore, evaluated separately from the SAI. To address the issue of potential collinearity, we re-calculated the SAI after removing the MedDietScore from its components (modified SAI: 0–9), and we repeated the analyses. Sensitivity analyses were performed to assess robustness. More specifically, local food consumption was categorized into quartiles and treated as a continuous variable as well. Associations with Mediterranean diet adherence and the SAI were examined using linear regression models adjusted for age, sex, smoking, and MoCA. All statistical analyses were performed using the SPSS software, version 29.0 (IBM Statistics, Athens, Greece).

## 3. Results

In Table 1, several sociodemographic, anthropometrical, lifestyle, dietary, clinical characteristics and the level of healthy aging, as derived from SAI index, of the 449 participants, categorized by the tertiles of local food consumption, are presented. Among the three groups studied [i.e., High consumption (≥45% of total food intake from local products), Moderate consumption (27–44%), and Low consumption (≤26%)], comparisons revealed that older adults with “High consumption” of local foods were more likely to be females (*p* < 0.001), have higher BMI (*p* = 0.006) but smaller waist-to-hip ratio (*p* = 0.006), to walk more times per week (*p* < 0.001), to not be smokers (*p* < 0.001), and to have higher adherence to the Mediterranean diet (*p* < 0.001) and higher levels of SAI (*p* = 0.003) compared to the other two food consumption groups.

Since the percentage of local food consumption was significantly correlated with both MedDietScore (rho = 0.337, *p* < 0.001) and SAI (rho = 0.162, *p* < 0.001), and given the potential for residual confounding in the observed associations between local food consumption groups and level of adherence to the Mediterranean diet but also with SAI levels, multiple adjusted linear regression models were employed to further investigate the proposed research hypothesis.

As shown in Table 2, after adjustments for age, sex, smoking habits, and cognitive status (i.e., MOCA), high local food consumption was associated with higher SAI levels (b = 0.817, SE = 0.189, 95% CI: 0.446 to 1.189, *p* < 0.001) compared to the low local food consumption group. This means that older adults whose diet consists of more than 45% local foods have an increase of 0.817 units in the SAI index. Additionally, high local food consumption was also associated with higher SAI levels (b = 0.493, SE = 0.18, 95% CI: 0.139 to 0.847, *p* = 0.007) compared to the “moderate” group. No statistically significant association was observed between “moderate” and “low” local food consumption groups in SAI levels (*p* = 0.133).

When we re-calculated the SAI, after removing the MedDietScore from its components (modified SAI: 0–9), both “high” vs. “moderate”, and “high” vs. “low” local food consumption remained significantly associated with higher modified SAI values (b = 0.328 ± SE: 0.163, 95% CI: 0.008 to 0.649, *p* = 0.045 and b = 0.573 ± SE: 0.173, 95% CI: 0.233 to 0.913, *p* = 0.001, respectively). However, when the MedDietScore was included as a covariate in the full regression model (Model 5), the association between local food consumption and modified SAI was attenuated and no longer reached statistical significance (*p* = 0.424 and *p* = 0.072, respectively) (Supplementary Table S1).

Accordingly, Table 3 indicates that after controlling for age, sex, walking duration, BMI, smoking habits, financial status, years of education, GDS, MOCA, and CVD risk factors, high local food intake was associated with higher adherence to the Mediterranean diet (b = 2.773, SE = 0.471, 95% CI: 1.845; 3.702, *p* < 0.001), in comparison to the low local food consumption group. Likewise, high local food intake was associated with higher MedDietScore (b = 1.941, SE = 0.442, 95% CI: 1.070; 2.812, *p* < 0.001) compared to the “moderate” group. In other words, older individuals whose diet comprises over 45% local foods perceive an increase of approximately 2.80 and 1.95 units in the MedDietScore, compared to “low” and “moderate” local food older adult consumers. No statistically significant associations were observed in the fully adjusted models in the comparison between the “moderate” and “low” groups (*p* = 0.097); however, in the crude model, it was found that older adults of the “moderate” group had an approximately 1-unit higher MedDietScore than those of the “low” group (*p* = 0.021). The robustness of our results was confirmed through sensitivity analyses (Supplementary Tables S2 and S3).

**Figure 1:**
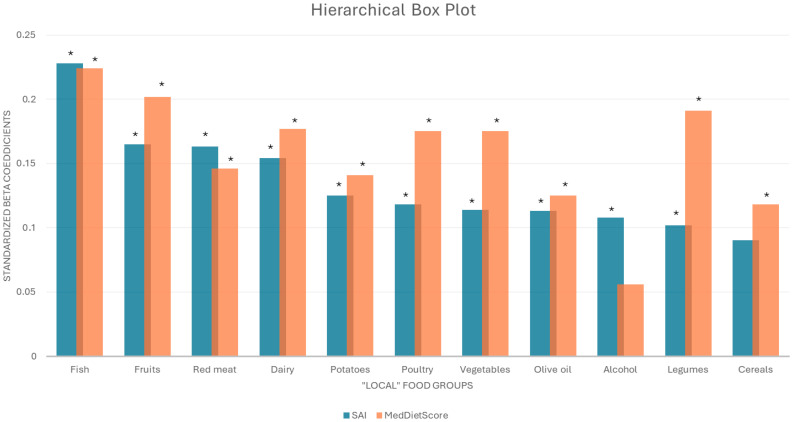
Hierarchical box plot depicting the associations of the eleven Mediterranean food groups with healthy aging (SAI) (blue columns) and Mediterranean diet adherence (MedDietScore) (orange columns), analyzed in the context of local food consumption. Results are presented as standardized beta coefficients from linear regression. Blue columns: Standardized beta coefficients for each local food group/dependent outcome: SAI/adjustments for age, sex, smoking habits, MoCA. Orange columns: Standardized beta coefficients for each local food group/dependent outcome: MedDietScore/adjustments for age, sex, BMI, walking, smoking, financial status, education, GDS, MoCA, CVD risk factors. SAI: Successful Aging Index; BMI: Body Mass Index; MOCA: Montreal Cognitive Assessment; GDS: Geriatric Depression Scale. *: *p*-Value < 0.05.

In Figure 1, the association between the eleven Mediterranean diet food groups, analyzed in the context of the proportion of local food consumption and healthy aging (blue boxes) or level of adherence to the Mediterranean diet (orange boxes), are depicted. Regarding healthy aging, it was revealed that local fish, followed by local fruits, red meat, dairy, potatoes, poultry, vegetables, olive oil, alcohol, and legumes were positively associated with SAI (all *p*’s < 0.05). Only for local cereals, there was no statistically significant association with SAI (*p* > 0.05). As far as MedDietScore was concerned, local fish, followed by fruits, legumes, equally by poultry and vegetables, red meat, potatoes, olive oil, and cereals were positively associated with MedDietScore (all *p*’s < 0.05). Only for local alcohol, there was no statistically significant association with MedDietScore (*p* > 0.05).

## 4. Discussion

The present work evaluated the association between local food consumption and both healthy aging and level of adherence to the Mediterranean diet, among older adults permanently living in the Peloponnese region. It was found that individuals who consumed local foods in more than 45% of their total diet were more likely to be females, have a higher BMI but lower waist-to-hip ratio, and demonstrate healthier lifestyle characteristics. Furthermore, high consumption of local food products (over 45% of total dietary intake) was significantly associated with greater adherence to the Mediterranean diet and higher levels of healthy aging. Hierarchically, local fish, followed by local fruits, red meat, dairy, potatoes, poultry, vegetables, olive oil, alcohol, and legumes were positively associated with healthy aging, while local fish, followed by fruits, legumes, equally by poultry and vegetables, red meats, potatoes, olive oil and cereals were positively associated with the level of adherence to the Mediterranean diet. These associations remained significant even after adjustments for several potential confounders, suggesting that food locality, and to an even greater extent, certain specific local foods, may play an important role in shaping health trajectories and quality of life of older Peloponnesians.

A noteworthy finding of this study is that high consumption of local foods was observed mainly among women. This may be indicative of gender disparities in dietary habits [26]. Although older women often have a central role in food choice, preparation, and procurement [27], additional factors may also be relevant. Previous studies have shown that women generally demonstrate greater health consciousness (e.g., they tend to consume red meat or processed foods less often) and higher engagement with diet to maintain health [28,29]. Cultural expectations and social norms may further reinforce women’s involvement in household food decisions and shape attitudes toward local foods [27,29], which may explain the greater adherence to local products observed in the present work.

Another interesting finding of this study is that the participants with higher consumption of local foods reported longer weekly walking durations and significantly lower smoking rates, i.e., a healthier lifestyle. This observation aligns with other studies like that of Monterossa et al., suggesting that local food consumption is not an isolated dietary practice but rather part of a broader pattern of healthy choices and attitudes toward health and other socio-economic influences [30]. It could be suggested that individuals who choose local foods may be more conscious about food quality and origin, adopt healthier habits, and generally demonstrate greater awareness of disease prevention. In this context, health “habitus” may partly explain all the observed benefits on healthy aging and adherence to the Mediterranean diet among high local food consumers. “Habitus” in sociology refers to how people perceive and respond to their social environment through personal habits, abilities, and character dispositions [31,32]. Thus, the choice of local foods may reflect not only food preferences, but it could be the result of health habitus, which may encompass certain attitudes and practices toward tradition, food quality, and health maintenance [31,32].

On the other hand, it should be noted that a higher BMI was observed among individuals who consumed a greater proportion of local products. As there is a lack of studies specifically examining the association between food locality and BMI, it could be hypothesized that the local foods consumed in the Peloponnese may reflect the characteristics of the Mediterranean diet. A relevant study by Trichopoulou et al., which included 23,597 participants, found that adherence to the Mediterranean dietary pattern was not associated with BMI [33]. It is well-known that the Mediterranean diet is characterized by high whole grain/cereal, fruit, vegetable, legume, potato and olive oil consumption; regular fish consumption; moderate consumption of dairy, eggs and poultry; limited consumption of red meat; and modest amounts of wine, typically consumed during meals. This dietary pattern, includes energy-dense items such as olive oil [7], which may contribute to increased body weight without negatively affecting overall diet quality [34]. Although the present study did not analyze the specific types of local foods consumed, this may explain the observed BMI levels. Interestingly, the waist-to-hip ratio was found to be lower in the group with high local food consumption compared to the other groups. This suggests that despite the higher BMI, individuals in this group may have a more favorable body fat distribution and a probably lower risk of developing metabolic complications [20]. However, the paradoxical finding of higher BMI but lower waist-to-hip ratio among high local food consumers may also reflect differences in body fat distribution among older Peloponnesian women. This explanation is in accordance with the findings of the BioCycle Study which was conducted in the state of New York. It was reported that women who adhere the most to the Mediterranean diet have lower regional adiposity than less adherent women [35].

Another remarkable finding is that only high (above 45% of the total dietary intake), and not moderate (26–44%), consumption of local products was associated with higher adherence to the Mediterranean diet and healthy aging, compared to low consumption. More specifically, consuming local foods > 45% of the total dietary intake was found to be associated with over 5% increase in the level of adherence to the Mediterranean diet (almost 3/55 increase in MedDietScore). This is a very important finding for public health authorities, since higher adherence to this dietary pattern has been already associated with the prevention and better management of age-related diseases such as CVD, metabolic disorders, frailty, etc. [6]. Accordingly, “high” consumption of local foods was associated with almost a 1/10 unit increase in healthy aging status, meaning longer maintenance of the functionality that enables higher quality of life in older age [17]. The absence of statistically significant differences between moderate and low consumption suggests possible thresholds in the observed associations. This means that a sufficient level of local food consumption may be required to achieve the required benefits. These dose-dependent associations, although not fully yet investigated, have been observed in the context of the Mediterranean diet. More specifically, high adherence to the Mediterranean diet is required to achieve reduction in risk frailty and pre-frailty [36], as well as an adherence of 4/18 to the Mediterranean diet for inflammation and cognition protection [37] in older adults. Moreover, regarding healthy aging (a complex and multifactorial concept), the consumption of local products, which often reflects the principles of the traditional Mediterranean diet, may support the findings through increased intake of antioxidants, polyphenols, and fibers [38,39,40], as well as through association with a healthier overall lifestyle [41]. Therefore, the regular and high percentage incorporation of local foods into the diets of older people may be a realistic nutritional strategy for promoting healthy aging in local populations.

To the best of our knowledge, this is the first study to evaluate the association of local food intake with healthy aging and the level of adherence to the Mediterranean diet among Peloponnesian older adults, and comparisons with other studies are limited. Nevertheless, the results of the current study should be evaluated considering the underlying limitations. Due to its observational design, causal relationships are prohibited. Recall bias may also exist in self-reported questionnaires, especially in dietary self-reported questionnaires which are prone to inconsistencies [42]. Moreover, while there is no universally accepted definition of healthy aging and the SAI index may not encompass all relevant domains, it remains a validated tool that has been applied in other aging-related studies [38,43]. Finally, since there are many definitions regarding food locality [44], the definition in this work was limited to products originating from the region of the Peloponnese. This area, a 21,549.6 km^2^ peninsula in southern mainland Greece, represents a culturally and agriculturally distinct region. This distinctiveness limits the generalizability of the findings, not only to other populations but also to younger age groups, since the sample consisted exclusively of older Peloponnesians and no comparable studies have yet been conducted.

## 5. Conclusions

To conclude, the findings of this work indicate that higher consumption of local foods, and more specifically consuming local foods in more than 45% of the total dietary intake, is favorably associated with greater adherence to the Mediterranean diet and better healthy aging. Additionally, older individuals with high local food consumption demonstrated healthier lifestyle habits, including longer walking duration and a lower prevalence of smoking. Moreover, those who consumed greater quantities of local foods as a percentage of their total diet were more likely to be females. The results suggest that food locality is not only a component of cultural identity, but it may also play an important role in shaping healthy dietary habits, health trajectories, and quality of life of older Peloponnesians. Future studies focusing on other Mediterranean regions could be useful so that our findings could be generalized to other populations. Moreover, it would be interesting to conduct research on families to better understand the gender differences observed in the present work and women’s family consumption food-decision-making patterns. In that way, public health authorities could more effectively promote healthy aging and sustainable food systems.

## Figures and Tables

**Table 1:** Sociodemographic, anthropometric, lifestyle, dietary, and clinical characteristics of the participants based on the local food consumption tertiles (High, Moderate, Low).

Characteristics	Overall (N = 449)	1st Tertile High Consumption (n = 150)	2nd Tertile Moderate Consumption (n = 152)	3rd Tertile Low Consumption (n = 147)	*p*-Value
Age (yrs)	71 (9.0)	71 (7.0)	72 (10)	72 (9.0)	0.498
Females, n (%)	266 (59)	110 (73.8) *^,†^	91 (59.7) *	67 (45.3)	**<0.001**
BMI (kg/m^2^)	29.1 (7.1)	30.1 (6.8) ^†^	29.5 (7.5) *	28 (7.2)	**0.006**
Waist/Hip ratio	0.93 (0.14)	0.91 (0.11) ^†^	0.93 (0.12) *	0.96 (0.17)	**0.006**
Smokers, n (%yes)	94 (21)	15 (10) *^,†^	32 (20.8) *	46 (31.2)	**<0.001**
Financial status (%low/medium/good/very good)	24.3/37.8/34.3/3.5	30.7/32.1/31.4/5.8	19.6/44.1/34.3/2.1	23.7/37.4/36/ 2.9	0.140
Years of education	12 (9.0)	12 (9.0)	12 (9.0)	12 (8.0)	0.310
Walking time per week (min)	150 (240)	180 (360) *^,†^	140 (240)	150 (270)	**<0.001**
GDS (0–15)	3.0 (5.0)	3.0 (4.0)	3.0 (5.0)	4.0 (4.0)	0.074
MOCA (0–30)	23 (7.0)	24 (7.0)	23 (6.0)	23 (6.5)	0.600
MedDietScore (0–55)	34 (5.0)	36 (5.0) *^,†^	33 (6.0) *	33 (5.0)	**<0.001**
CVD risk factors (0–4)	2.0 (2.0)	2.0 (2.0)	2.0 (2.0)	2.0 (2.0)	0.860
SAI (0–10)	4.5 (2.2)	4.8 (2.4)*^,†^	4.2 (2.2)	4.4 (2.0)	**0.003**

Results are presented as median and interquartile ranges for continuous variables or frequencies and percentages for categorical variables. *p*-values derived from Kruskal–Wallis H test and chi-square test for categorical data. BMI: Body Mass Index; GDS: Geriatric Depression Scale; MOCA: Montreal Cognitive Assessment; SAI: Successful Aging Index. * *p*-value < 0.05 for the comparisons vs. Moderate consumption group; ^†^ *p*-value < 0.05 for the comparisons vs. Low consumption group; bold *p*’s indicate statistically significant differences.

**Table 2:** Results from multiple linear regression models performed to evaluate the association of local food consumption groups (i.e., “High vs. Low”, “High vs. Moderate”, “Moderate vs. Low”) with the healthy aging level.

Dependent Outcome	Models	b ± SE (for Local Food Consumption)	95% CI	*p*-Value
[SAI (0–10)]	Model 1: High vs. Low local food consumption	0.618 ± 0.181	0.261; 0.974	<0.001
	Model 2: Model 1 + age, sex	0.752 ± 0.188	0.382; 1.121	<0.001
	Model 3: Model 2 + smoking habits	0.751 ± 0.195	0.367; 1.136	<0.001
	Model 4: Model 3 + MOCA	0.817 ± 0.189	0.446; 1.189	<0.001
	Model 1: High vs. Moderate local food consumption	0.505 ± 0.180	0.150; 0.859	0.005
	Model 2: Model 1 + age, sex	0.497 ± 0.180	0.144; 0.850	0.006
	Model 3: Model 2 + smoking habits	0.497 ± 0.183	0.137; 0.857	0.007
	Model 4: Model 3 + MOCA	0.493 ± 0.180	0.139; 0.847	0.007
	Model 1: Moderate vs. Low local food consumption	0.113 ± 0.164	−0.209; 0.435	0.491
	Model 2: Model 1 + age, sex	0.183 ± 0.164	−0.140; 0.506	0.266
	Model 3: Model 2 + smoking habits	0.196 ± 0.166	−0.130; 0.522	0.238
	Model 4: Model 3 + MOCA	0.241 ± 0.160	−0.074; 0.556	0.133

Results are presented as standardized b-coefficient (b), standard error (SE) and 95% CI: 95% Confidence Interval. SAI: Successful Aging Index; MOCA: Montreal Cognitive Assessment. Level of statistical significance was set at *p*-value < 0.05.

**Table 3:** Results from multiple linear regression models performed to evaluate the association of local food consumption groups (i.e., “High vs. Low”, “High vs. Moderate”, “Moderate vs. Low”) with the level of adherence to the Mediterranean diet.

Dependent Outcome	Models	b ± SE (for Local Food Consumption Group)	95% CI	*p*-Value
MedDietScore (0–55)	Model 1: High vs. Low local food consumption	3.300 ± 0.421	2.470; 4.129	<0.001
	Model 2: Model 1 + age, sex	2.904 ± 0.437	2.045; 3.764	<0.001
	Model 3: Model 2 + Walking time, BMI, smoking habits	2.816 ± 0.465	1.899; 3.732	<0.001
	Model 4: Model 3 + financial status, education	2.909 ± 0.460	2.003; 3.815	<0.001
	Model 5: Model 4 + MOCA, GDS	2.731; 0.469	1.807; 3.655	<0.001
	Model 6: Model 5 + CVD risk factors	2.773 ± 0.471	1.845; 3.702	<0.001
	Model 1: High vs. Moderate local food consumption	2.274 ± 0.419	1.450; 3.099	<0.001
	Model 2: Model 1 + age, sex	2.167 ± 0.423	1.333; 3.001	<0.001
	Model 3: Model 2 + walking time, BMI, smoking habits	2.087 ± 0.433	1.235; 2.938	<0.001
	Model 4: Model 3 + financial status, education	1.947 ± 0.439	1.081; 2.812	<0.001
	Model 5: Model 4 + MOCA, GDS	1.943 ± 0.442	1.073; 2.813	<0.001
	Model 6: Model 5 + CVD risk factors	1.941 ± 0.442	1.070; 2.812	<0.001
	Model 1: Moderate vs. Low local food consumption	1.025 ± 0.440	0.159; 1.892	0.021
	Model 2: Model 1 + age, sex	0.849 ± 0.446	−0.028; 1.726	0.058
	Model 3: Model 2 + walking time, BMI, smoking habits	0.599 ± 0.467	−0.322; 1.520	0.201
	Model 4: Model 3 + financial status, education	0.842 ± 0.451	−0.046; 1.729	0.063
	Model 5: Model 4 + MOCA, GDS	0.801 ± 0.464	−0.112; 1.714	0.085
	Model 6: Model 5 + CVD risk factors	0.777 ± 0.466	−0.141; 1.695	0.097

Results are presented as standardized b-coefficient (b), standard error (SE) and 95% CI: 95% Confidence Interval. BMI: Body Mass Index; MOCA: Montreal Cognitive Assessment; GDS: Geriatric Depression Scale. Level of statistical significance was set at *p*-value < 0.05.

## Data Availability

The data presented in this study are available on request from the corresponding author due to privacy and ethical reasons.

## Supplementary Materials

The following supporting information can be downloaded at: https://www.mdpi.com/article/10.3390/nu17182975/s1, Table S1: Results from multiple linear regression models performed to evaluate the association of local food consumption groups (i.e., “High vs. Low”, “High vs. Moderate”, “Moderate vs. Low”) with the modified healthy aging level (i.e., modified SAI).; Table S2: Results from univariate linear regression analyses evaluating the association of local food consumption groups (in quartiles, Q1: < 22.2 %, Q2: 22.2–34.7 %, Q3: 34.7–52.8, Q4: ≥ 52.8) with healthy aging (SAI).; Table S3: Results from linear regression models evaluating the association of local food consumption (continuous variable) with healthy aging (SAI).

## Abbreviations

The following abbreviations are used in this manuscript:

BMI	Body Mass Index
CVD	Cardiovascular Disease
GDS	Geriatric Depression Scale
MoCA	Montreal Cognitive Assessment
SAI	Successful Aging Index
