# Supplementary material for: Higher Local Food Consumption Is Associated with Higher Adherence to the Mediterranean Diet and Better Healthy Aging: Results of the DIAPELH Study
Source: Nutrients. 2025 Sep 17;17(18):2975. doi: 10.3390/nu17182975 (PMC12473110; doi:10.3390/nu17182975)
Supplement: Supplementary file 1 [file nutrients-17-02975-s001.zip › nutrients-3863567-supplementary.pdf]

Supplementary Material

Supplementary Table S1. Results from multiple linear regression models performed to evaluate the association of local food consumption groups (i.e., "High vs. Low", "High vs. Moderate", "Moderate vs. Low") with the modified healthy aging level (i.e., modified SAI).

Dependent outcome	Models	b ± SE (for local food consumption)	95% CI	p-value
[modified SAI (0 – 9)]	Model 1: *High vs. Low* local food consumption	0.326 ± 1.67	-0.002; 0.654	0.052
	Model 2: Model 1 + age, sex	0.499 ± 0.171	0.162; 0.836	0.004
	Model 3: Model 2 + smoking habits	0.514 ± 0.178	0.164; 0.864	0.004
	Model 4: Model 3 + MOCA	0.573 ± 0.173	0.233; 0.913	0.001
	Model 5: Model 4 + MedDietScore	0.324 ± 0.179	-0.029; 0.678	0.072
	Model 1: *High vs. Moderate* local food consumption	0.304 ± 0.163	-0.018; 0.625	0.064
	Model 2: Model 1 + age, sex	0.313 ± 0.162	-0.006; 0.632	0.055
	Model 3: Model 2 + smoking habits	0.329 ± 0.165	0.003; 0.654	0.048
	Model 4: Model 3 + MOCA	0.328 ± 0.163	0.008; 0.649	0.045
	Model 5: Model 4 + MedDietScore	0.131 ± 0.163	-0.191; 0.453	0.424
	Model 1: *Moderate vs. Low* local food consumption	0.022 ± 0.149	-0.272; 0.316	0.883
	Model 2: Model 1 + age, sex	0.111 ± 0.149	-0.182; 0.404	0.455
	Model 3: Model 2 + smoking habits	0.131 ± 0.150	-0.165; 0.427	0.384
	Model 4: Model 3 + MOCA	0.171 ± 0.144	-0.113; 0.455	0.236
	Model 5: Model 4 + MedDietScore	0.106 ± 0.141	-0.171; 0.383	0.454

Results are presented as standardized b-coefficient (b), standard error (SE) and 95% CI: 95% Confidence Interval and p-values. Modified SAI: Successful Aging Index (excluding the MedDietScore component); MOCA: Montreal Cognitive Assessment

Supplementary Table S2. Results from univariate linear regression analyses evaluating the association of local food consumption groups (in quartiles, Q1: < 22.2 %, Q2: 22.2 – 34.7 %, Q3: 34.7 – 52.8, Q4: ≥ 52.8) with healthy aging (SAI).

Dependent outcome	Models	b ± SE (for local food consumption)	95% CI	p-value
SAI (0 – 10)	Q4 *	ref		
	Q3 *	-0.600 ± 0.192	-0.977; -0.223	**0.002**
	Q2 *	-0.882 ± 0.196	-1.267; -0.497	**< 0.001**
	Q1 *	-0.822 ± 0.210	-1.235; -0.410	**< 0.001**
MedDietScore (0-55)	Q4 **	ref		
	Q3 **	-1.223 ± 0.496	-2.199; -0.247	**0.014**
	Q2 **	-2.674 ± 0.506	-3.668; -1.680	**< 0.001**
	Q1 **	-2.671 ± 0.551	-3.755; 1.588	**< 0.001**

Results are presented as standardized b-coefficient (b), standard error (SE), 95% CI: 95% Confidence Interval and p-values.

*: Adjusted for age, sex, smoking, MoCA

** Adjusted for: age, sex, bmi, smoking, walking time, education, financial status, GDS, MoCA, CVD risk factors.

BMI: Body Mass Index; GDS: Geriatric Depression Scale; MOCA: Montreal Cognitive Assessment; SAI: Successful Aging Index.

Supplementary Table S3. Results from linear regression models evaluating the association of local food consumption (continuous variable) with healthy aging (SAI).

Dependent outcome	Models	b ± SE (for local food consumption)	95% CI	p-value
SAI (0 – 10)	Model 1: Local food consumption	0.014 ± 0.004	0.007; 0.021	**< 0.001**
	Model 2: Model 1 + age, sex	0.015 ± 0.004	0.008; 0.022	**< 0.001**
	Model 3: Model 2 + smoking habits	0.015 ± 0.004	0.008; 0.023	**< 0.001**
	Model 4: Model 3 + MOCA	0.016 ± 0.004	0.009; 0.023	**< 0.001**
MedDietScore (0-55)	Model 1: Local food consumption	0.066 ± 0.009	0.048; 0.083	**<0.001**
	Model 2: Model 1 + age, sex	0.06 ± 0.009	0.043; 0.078	**< 0.001**
	Model 3: Model 2 + Walking time, BMI, smoking habits	0.055 ± 0.009	0.037; 0.073	**< 0.001**
	Model 4: Model 3 + financial status, education	0.057 ± 0.009	0.038; 0.075	**< 0.001**
	Model 5: Model 4 + MOCA, GDS	0.055 ± 0.01	0.037; 0.074	**< 0.001**
	Model 6: Model 5 + CVD risk factors	0.056 ± 0.01	0.037; 0.074	**< 0.001**

Results are presented as standardized b-coefficient (b), standard error (SE), 95% CI: 95% Confidence Interval and p-values. SAI: Successful Aging Index; MOCA: Montreal Cognitive Assessment
